# Effect of bariatric surgery on carotid intima-media thickness: A meta-analysis based on observational studies

**DOI:** 10.3389/fsurg.2022.1068681

**Published:** 2023-01-10

**Authors:** Hui Zhou, Yangli Jin, Senjie Dai, Chenglong Dai, Xia Ye

**Affiliations:** ^1^ Department of Ultrasound, The Affiliated Hospital of Ningbo University, LiHuiLi Hospital, Ningbo, China; ^2^ Department of Ultrasound, Ningbo Yinzhou No. 2 Hospital, Ningbo, China; ^3^ The Second Clinical Medical College, Zhejiang Chinese Medical University, Hangzhou, China; ^4^ School of Medical Imaging, Hangzhou Medical College, Hangzhou, China; ^5^ General Family Medicine, Ningbo Yinzhou No. 2 Hospital, Yinzhou district, ningbo, China

**Keywords:** bariatric surgery, carotid intima-Media thickness, obesity, weight loss, meta-analysis

## Abstract

**Objective:**

This meta-analysis aimed to investigate the effect of bariatric surgery on CIMT in people with obesity.

**Methods:**

PubMed, Web of Science, Embase, and the Cochrane Library were searched for observational studies assessing the effect of bariatric surgery on CIMT from inception to August 2022. Mean difference (MD) and 95% confidence intervals were calculated to assess CIMT.

**Results:**

A total of 23 studies, including 1,349 participants, were eligible to participate in this meta-analysis. The results revealed that CIMT was significantly decreased at 6 months, 12 months, and more than 18 months after bariatric surgery compared with baseline (6 months: MD = 0.09; *P *< 0.01; 12 months: MD = 0.12; *P *< 0.01; more than 18 months: MD = 0.14; *P *= 0.02). Meanwhile, laparoscopic Roux-en-Y gastric bypass (LRYGB) seemed to be more effective than laparoscopic sleeve gastrectomy (LSG) in lowering CIMT in terms of the type of surgery (LSG: MD = 0.11; *P *< 0.01; LRYGB: MD = 0.14; *P *< 0.01). Lastly, the benefits of bariatric surgery on CIMT was independent of gender (Male: MD = 0.06; *P *= 0.04; Female: MD = 0.08; *P *= 0.03).

**Conclusions:**

Bariatric surgery is consistently effective in reducing CIMT in people with obesity.

## Introduction

The prevalence of obesity has increased worldwide over the last decades and has emerged as one of the greatest public health challenges (1). Obesity poses many health problems, and there is currently a huge body of clinical evidence linking obesity to cardiovascular diseases (2, 3). On the one hand, obesity can directly adapt to overweight by inducing changes in the cardiovascular structure and functions; on the other hand, obesity can induce cardiovascular risk factor conditions such as hyperlipidemia, hyperglycemia and insulin resistance (4–6).

Carotid intima-media thickness (CIMT), measured by ultrasonography, is a noninvasive, rapid, reproducible marker of subclinical atherosclerosis that is positively associated with the risk of cardiovascular events and is widely considered to be an independent predictor of cardiovascular events (7, 8). Bariatric surgery has become a promising option for weight loss in situations where diet, lifestyle changes, and medical treatment do not produce the desired results (9, 10). At the same time, the benefits of bariatric surgery for weight loss as well as metabolic improvement have been established; nevertheless, its efficacy on CIMT remains to be validated. Currently, an increasing number of studies have explored the effect of bariatric surgery on CIMT in people with obesity. Therefore, this meta-analysis aimed to investigate the effect of bariatric surgery on alterations in CIMT in people with obesity by reviewing relevant studies.

## Methods

### Search strategy

This meta-analysis was conducted in accordance with the Preferred Reporting Items for Systematic Reviews and Meta-Analyses (PRISMA) reporting guidelines (11). Electronic databases, including PubMed, Web of Science, Embase, and the Cochrane Library, were independently searched by two authors for articles on the effect of bariatric surgery on carotid intimal thickness. All included articles were published before August 2022. Keywords used for the search were as follows: “carotid intima-media thickness” OR “ intima-media thickness” OR “carotid intima-media” OR “intima-media” OR “CIMT” OR “IMT” AND “bariatric procedure” OR “weight loss procedure” OR “bariatric surgery” OR “GB” OR “gastric bypass” OR “laparoscopic Roux-en-Y gastric bypass” OR “laparoscopic sleeve gastrectomy” OR “sleeve gastrectomy” OR “LRYGB” OR “SG” OR “LSG”. Furthermore, to avoid the omission of any additional qualifying articles, the references of the eligible articles were manually examined.

### Inclusion and exclusion criteria

Studies were included if they met the following criteria: (1) all participants were people with obesity; (2) participants underwent bariatric surgery; (3) the outcome was CIMT; (4) pre- and postoperative CIMT data were available.

Studies meeting any of the following criteria were excluded: (1) the article was not written in English; (2) no relevant or available data could be extracted; (3) in the event of duplicate or continuously updated publications, the latest edition was selected.

### Quality assessment and data extraction

The Newcastle-Ottawa Scale (NOS) checklist was employed to evaluate the quality of the included observational studies (OBSs) based on three aspects: patient selection, comparability of groups, and evaluation of outcomes (12). The checklist has a maximum score of 9 points, and articles scoring less than 6 points are considered low quality.

According to a predesigned data extraction form, two authors independently reviewed the included articles and extracted the relevant data. These data included study characteristics (author, year, country, follow-up time, and type of surgery) and patient characteristics (age, number of patients, body mass index (BMI), and levels of fasting plasma glucose, triglycerides, total cholesterol, high-density lipoprotein cholesterol (HDL-c), low-density lipoprotein cholesterol (LDL-c), etc.). Discrepancies were resolved by two reviewers in consultation with a third party.

### Statistical analysis

Statistical analysis of the data was performed using RevMan 5.3 software (The Cochrane Collaboration) and STATA 12.0. For some studies that provided only subgroup data but lacked combined results, we included subgroup data simultaneously for meta-analysis. Mean difference (MD) with 95% confidence intervals (CI) was used to compare CIMT before and after bariatric surgery. Considering the differences in surgical type and measurement site between studies, the random-effects model was applied for statistical analysis to improve the reliability of the results. Heterogeneity between the studies was determined using the Chi-square test and *I*^2^ statistics, and heterogeneity was considered significant when *I*^2^ was greater than 50%. Publication bias was assessed by using the Eggers' test. Meanwhile, sensitivity analyses were conducted to assess the reliability of the overall results. *P* < 0.05 was considered statistically significant.

## Results

### Study selection

**Figure 1:**
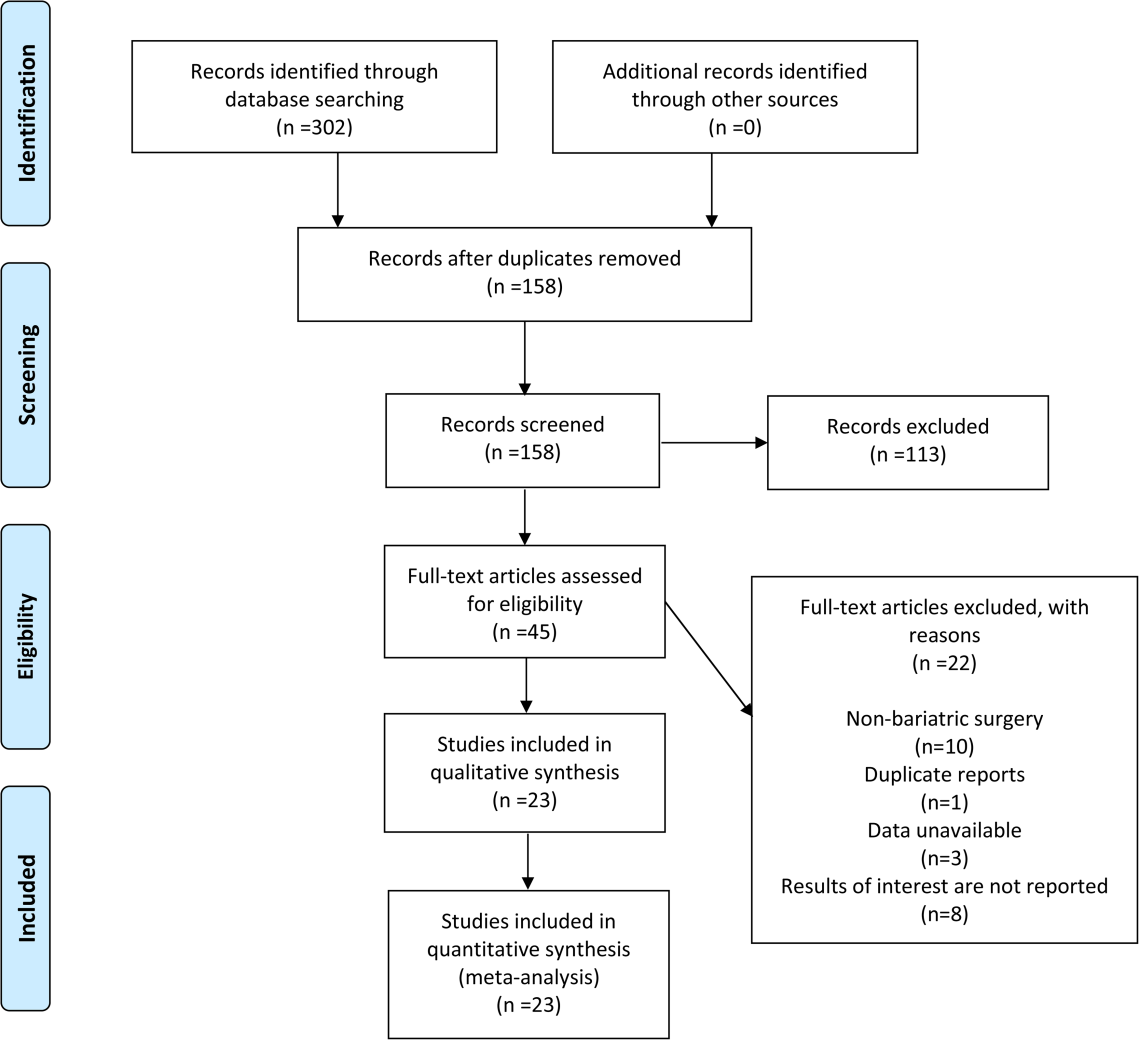
Flow chart for the selection of studies included in the meta-analysis.

According to the developed search strategy, 302 articles were retrieved from four databases, with no additional articles obtained through other channels. By reviewing the titles and abstracts, duplicated and irrelevant articles were excluded, yielding 45 articles for full-text review. A total of 22 studies were excluded due to non-bariatric surgery, duplicate reports, data unavailable and no relevant results, and 23 studies were finally included in this meta-analysis (13–35). The detailed PRISMA flowchart is displayed in Figure 1.

### Study characteristics and quality assessment

Table 1 summarizes the characteristics of the included studies. A total of 23 studies involving 1,349 participants who had undergone bariatric surgery were included. These 23 studies were published between 2009 and 2022 and were conducted in several countries (9 in Turkey, 2 in Austria, 2 in Brazil, 2 in Spain, 1 in Italy, 1 in China, 1 in Chile, 1 in the United States, 1 in the Netherlands, 1 in India, 1 in Iran, and 1 in Egypt). The follow-up time of the included studies ranged from 3 months to 60 months, with the majority having a follow-up period of 6 to 12 months. In addition, laparoscopic sleeve gastrectomy (LSG) and laparoscopic Roux-en-Y gastric bypass (LRYGB) were the primary surgeries performed for weight loss in these studies.

**Table 1:** Characteristics of all the studies included in the meta-analysis.

Author	Year	Country	No. of patients	Age	Follow-up (months)	Type of surgery
Altin	2018	Turkey	105	43.61 ± 12.24	6	LSG
Baykara	2018	Turkey	60	M:35.14 ± 11.30 F:38.86 ± 10.39	18	LSG
Borzì	2020	Italy	17	39.8 ± 10.4	16 ± 8	LAGB; GBP; BDP
Cekici	2021	Turkey	47	38 ± 10.48	6	LSG
Chen	2017	China	33	47.7 ± 11.6	12	LRYGB
Cobeta	2020	Spain	40	LSG:46 ± 9 LRYGB:51 ± 9	6	LSG; LRYGB
Elitok	2020	Turkey	23	40.4 ± 5.6	12	LRYGB
Elkan	2022	Turkey	44	37.2 ± 10.9	6	LSG
Garcia	2013	Chile	27	43.5 ± 8.8	12	LSG; LRYGB
Gómez-Martin	2020	Spain	40	LSG: 46 ± 9 LRYGB: 48 ± 9	12	LSG; LRYGB
Habib	2009	United States	22 28	44.5 ± 2.4 44.8 ± 1.8	12 24	LRYGB
Jonker	2018	Netherlands	166	42.5	12	LSG; LRYGB
Kaul	2021	India	40	40.8 ± 10.7	12	LSG; OAGB; LRYGB
Kaya	2021	Turkey	71	37.6 ± 11.2	6	LSG
Nabavi	2022	Iran	32	38.18 ± 1.18	6	LRYGB
Saleh	2012	Brazil	47	41	6-19	LRYGB
Salman	2021	Egypt	120	43.7 ± 8.5	12	LSG
Sarmento	2009	Brazil	18	44.1 ± 9.8	12	LRYGB
Solmaz	2016	Turkey	48	LSG:42.96 ± 7.87 LGP:38.3 ± 9.88	6	LSG; LGP
Sturm	2009	Austria	37	NA	18	LAGB; GBP
Tschoner	2013	Austria	52	35.3	60	LAGB; GBP
Yavuz	2021	Turkey	216	42.3 ± 10.1	12	NA
Yorulmaz	2016	Turkey	16	39.12 ± 10.63	4.6	LSG

M: male; F: female; LSG: laparoscopic sleeve gastrectomy; LRYGB: laparoscopic Roux-en-Y gastric bypass; GBP: gastric by-passes; BDP: biliopancreatic diversions; OAGB: one anastomosis gastric bypass; LGP: laparoscopic gastric plication; LAGB: laparoscopic adjustable gastric banding; NA: not available.

The detailed characteristics of the patients are presented in Table 2, including BMI, fasting blood glucose levels, total cholesterol levels, triglyceride levels, HDL-c levels, LDL-c levels, number of hypertensive patients, number of diabetic patients, number of hyperlipidemia patients, and number of smokers, all of them being risk factors for cardiovascular diseases.

**Table 2:** Characteristics of the patients included in the study.

Author	Year	BMI	FPG mg/dl	TC mg/dl	TGs mg/dl	HDL-c mg/dl	LDL-c mg/dl	HT	HL	DM	Somking
Altin	2018	46.95 ± 7.54	123.76 ± 35.21	217.33 ± 41.83	126.35 ± 57.65	46.91 ± 9.63	145.3 ± 35.49	47	78	28	26
Baykara	2018	47.55 ± 5.40	103.71 ± 27.61	206.55 ± 30.57	163.36 ± 85.17	39.41 ± 6.99	122.69 ± 29.92	NA	NA	NA	NA
Borzì	2020	50.4 ± 11.5	89 ± 13	193 ± 40	134 ± 48	48 ± 12	117 ± 34	4	NA	2	4
Cekici	2021	47.31 ± 6.10	103.74 ± 27.68	193.26 ± 33.21	233.45 ± 74.44	37.72 ± 12.70	108.43 ± 28.76	18	10	16	12
Chen	2017	30.9 ± 4.6	8.4 ± 2.4 mmol/L	4.9 ± 1.0 mmol/L	2.8 ± 2.5 mmol/L	1.0 ± 0.3 mmol/L	2.9 ± 1.0 mmol/L	18	NA	NA	NA
Cobeta	2020	LSG: 45.0 ± 6.9 LRYGB: 43.7 ± 7.2	107 ± 34 129 ± 60	183 ± 48 159 ± 49	154 ± 100 270 ± 48	40 ± 6 39 ± 9	117 ± 36 82 ± 29	10 18	8 14	6 10	5 6
Elitok	2020	52 ± 6.9	106 ± 28	NA	148 ± 70	41 ± 7	126 ± 33	NA	NA	NA	NA
Elkan	2022	47.1 ± 5.8	102 ± 17	193 ± 30	247 ± 69	35.1 ± 11.2	107 ± 28	16	NA	15	11
Garcia	2013	38 ± 4	105 ± 18	223 ± 46	231 ± 143	45 ± 9	136 ± 36	NA	NA	NA	NA
Gómez-Martin	2020	LSG: 43.0 ± 4.0 LRYGB: 47.4 ± 6.4	114.3 ± 33.8 111.3 ± 24.3	NA	NA	NA	NA	NA	NA	NA	NA
Habib	2009	12M: 46.7 ± 1.6 24M: 47.2 ± 1.4	NA	188 ± 10 189 ± 8	172 ± 24 168 ± 15	45 ± 2 52 ± 2	110 ± 8 102 ± 7	NA	NA	NA	NA
Jonker	2018	43.4 ± 4.8	NA	NA	NA	NA	NA	63	25	27	C:6 *P*:79
Kaul	2021	45.9 ± 6.5	NA	171 ± 32.7	124.2 ± 45.8	41.5 ± 8.2	103.8 ± 29.7	17	NA	15	8
Kaya	2021	47.7 ± 6.5	104.6 ± 23.2	NA	226 ± 72	40.8 ± 10.5	108 ± 27	23	NA	26	18
Nabavi	2022	43.66 ± 6.44	110.94 ± 45.59	171.62 ± 42.83	150.94 ± 81.82	NA	NA	7	20	10	5
Saleh	2012	47.1 ± 5.5	94.7 ± 21.7	183.4 ± 37.7	148.6 ± 94.6	40.4 ± 9.7	113.9 ± 29.2	29	11	NA	C:2 *P*:9
Salman	2021	43.8 ± 5.2	8.5 ± 3.52 mmol/L	5.48 ± 1.29 mmol/L	2.17 ± 0.52 mmol/L	1.17 ± 0.19 mmol/L	3.13 ± 1.08 mmol/L	48	35	49	NA
Sarmento	2009	44.3 ± 6.4	97.9 ± 29.6	NA	145.7 ± 72.7	51.9 ± 15.7	108.5 ± 33.6	10	6	3	NA
Solmaz	2016	LSG: 44.84 ± 3.63 LGP: 45.39 ± 3.69	NA	206.52 ± 49.74 210.61 ± 53.01	190.88 ± 151.27 177.52 ± 100.14	47.6 ± 11.73 46.48 ± 10.48	123.4 ± 40.98 130.7 ± 45.36	NA	NA	NA	NA
Sturm	2009	42.42 + 3.98	98.2 + 11.5	194.5 ± 40.8	120 ± 58	49.4 ± 10.2	120.1 ± 34.9	NA	NA	NA	NA
Tschoner	2013	43.6 + 4.9	5.54 + 0.62 mmol/L	4.94 ± 0.91 mmol/L	1.24 ± 0.77 mmol/L	1.25 ± 0.29 mmol/L	3.04 ± 0.83 mmol/L	31	NA	NA	NA
Yavuz	2021	48.25 ± 7.09	109.70 ± 47.42	206.20 ± 42.61	156.90 ± 92.62	47.62 ± 11.64	129.90 ± 41.45	NA	NA	125	NA
Yorulmaz	2016	NA	NA	209.12 ± 48.71	153.00 ± 61.05	45.125 ± 6.80	209.12 ± 48.71	NA	NA	NA	NA

BMI: body mass index; FPG: fasting plasma glucose; TC: total cholesterol; TGs: triglycerides; HDL-c: high-density lipoprotein cholesterol; LDL-c: low-density lipoprotein cholesterol; HT: hypertension; HL: hyperlipidemia; DM: diabetes mellitus; M: months; C: current; *P*: past; NA: not available.

The NOS checklist was utilized to evaluate the quality of the included OBSs. All included studies scored greater than 6 points and were of high quality, as listed in Supplementary Table S1.

### CIMT

**Figure 2:**
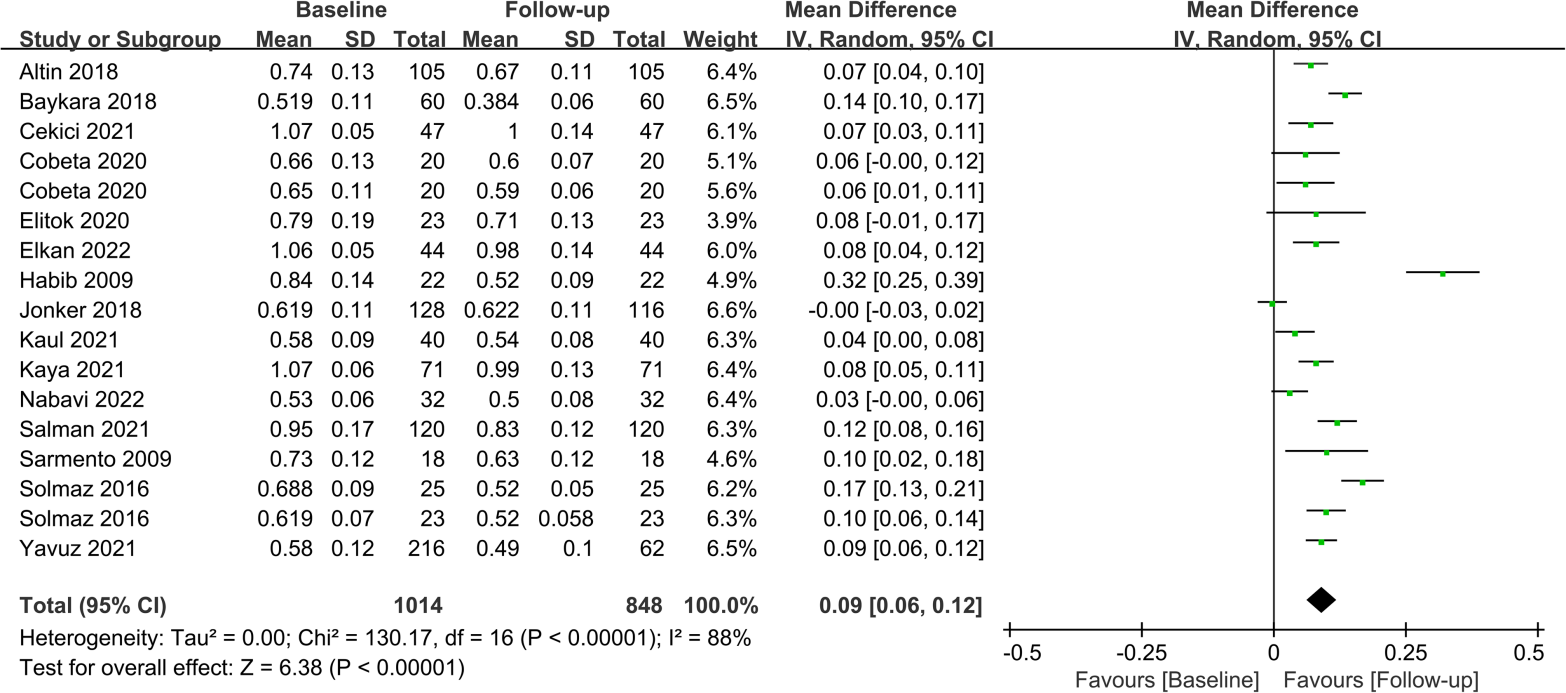
Forest plot of meta-analysis of CIMT at 6 months after bariatric surgery. (Cobeta 2020 and Solmaz 2016 respectively included two subsets of data for meta-analysis).

**Figure 3:**
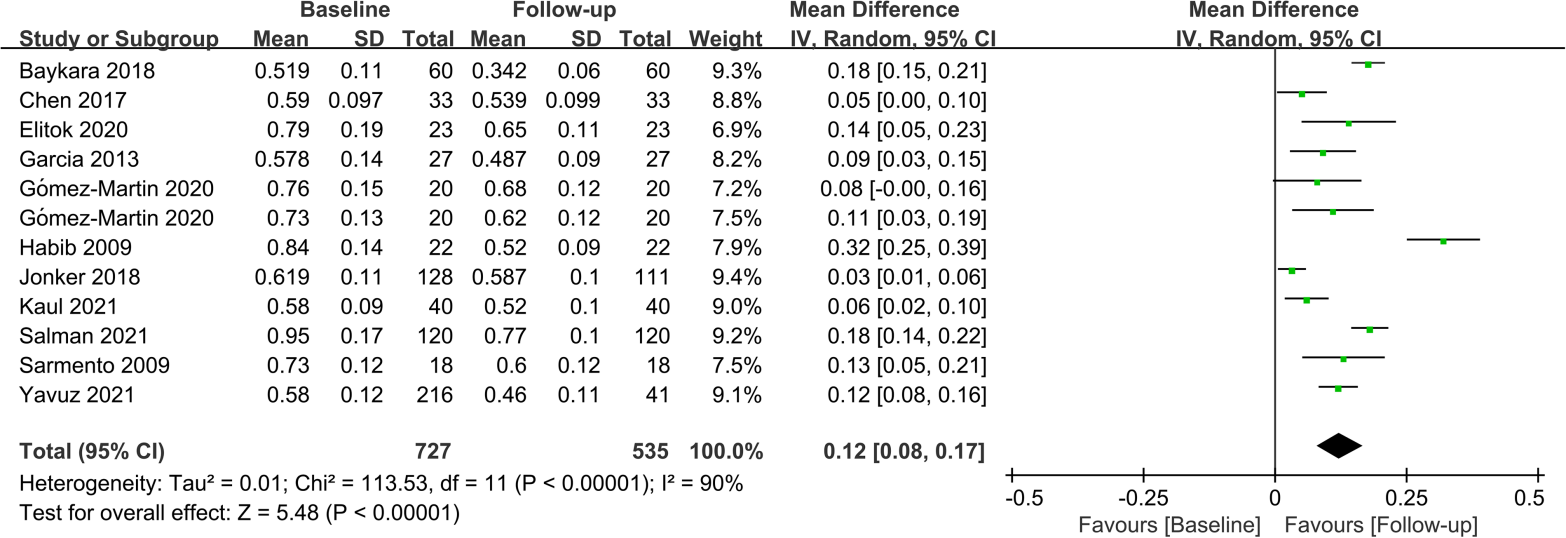
Forest plot of meta-analysis of CIMT at 12 months after bariatric surgery. (Gómez-Martin 2020 included two subsets of data for meta-analysis).

**Figure 4:**
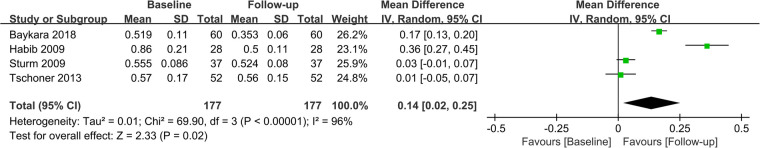
Forest plot of meta-analysis of CIMT more than 18 months after bariatric surgery.

Fifteen studies assessed CIMT at 6 months after the surgery, and combined results revealed a significant decrease in CIMT compared to baseline, with significant heterogeneity among the studies (MD = 0.09; *P *< 0.01; *I*^2 ^= 88%; Figure 2). A total of 11 studies investigated CIMT at 12 months after surgery, and there was high heterogeneity among the studies. The pooled results showed that CIMT was decreased by 0.12 mm from baseline, and the difference was statistically significant (MD = 0.12; *P *< 0.01; *I*^2 ^= 90%; Figure 3). Besides, four studies evaluated the longer-term effects of bariatric surgery on CIMT (follow-up of 18 months or more), and statistical analyses determined that CIMT was significantly lower (MD = 0.14; *P *= 0.02), as illustrated in Figure 4. Taken together, the benefits of decreased CIMT following bariatric surgery increased with the extension of follow-up time.

### Subgroup analysis

**Figure 5:**
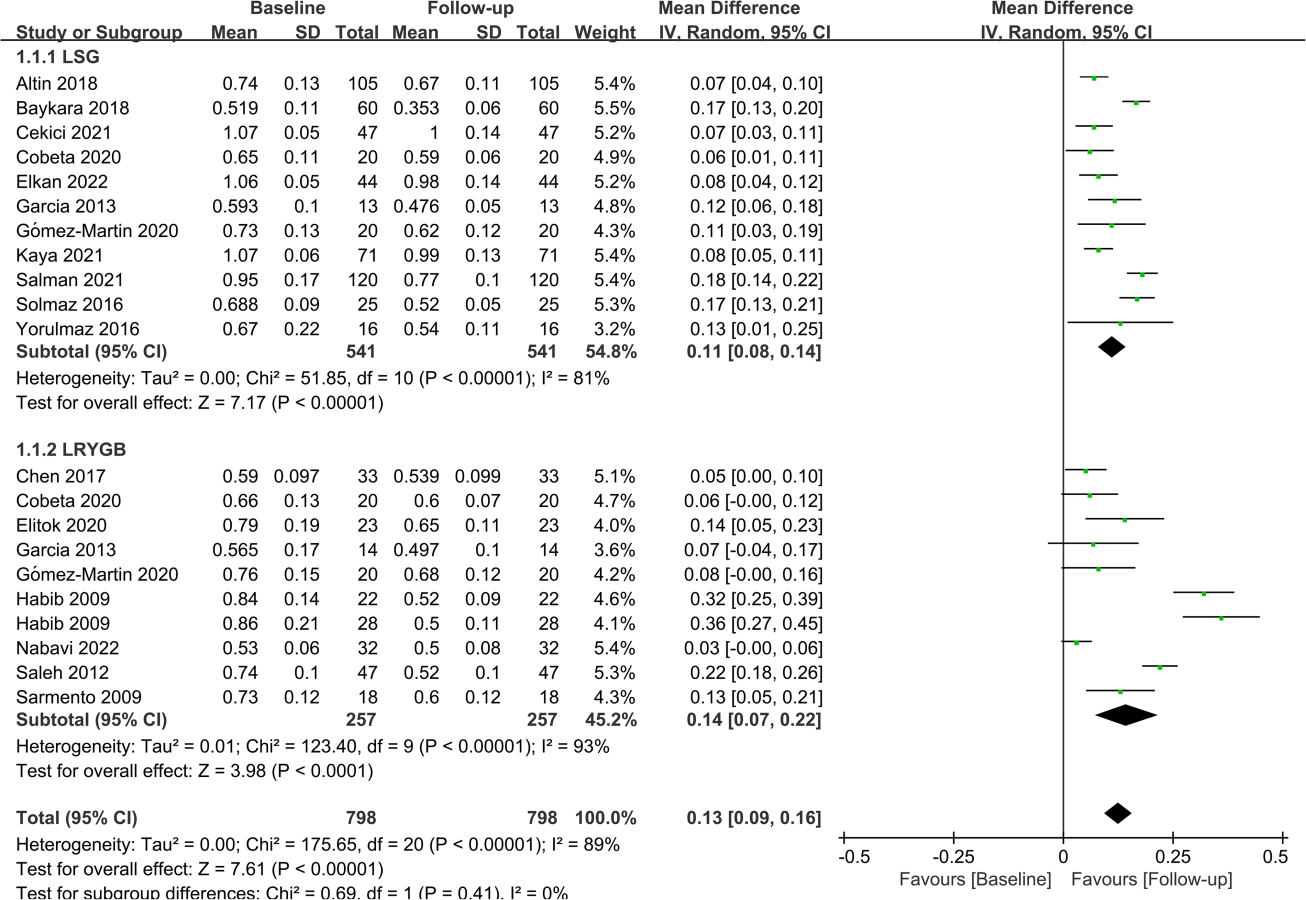
Forest plot of meta-analysis for CIMT by the type of surgery. (Cobeta 2020, Garcia 2013, Habib 2009, Gómez-Martin 2020 respectively included two subsets of data for meta-analysis).

Subgroup analyses were performed to evaluate the impact of different surgical procedures on CIMT based on their distinct characteristics. Eleven studies (*n* = 541) and nine studies (*n* = 798) investigated the effects of LSG and LRYGB on CIMT, respectively. Integrated data showed that both procedures significantly lowered CIMT in patients (LSG: MD = 0.11; *P *< 0.01; LRYGB: MD = 0.14; *P *< 0.01). At the same time, LRYGB seemed superior to LSG in reducing CIMT. The detailed results are displayed in Figure 5.

**Figure 6:**
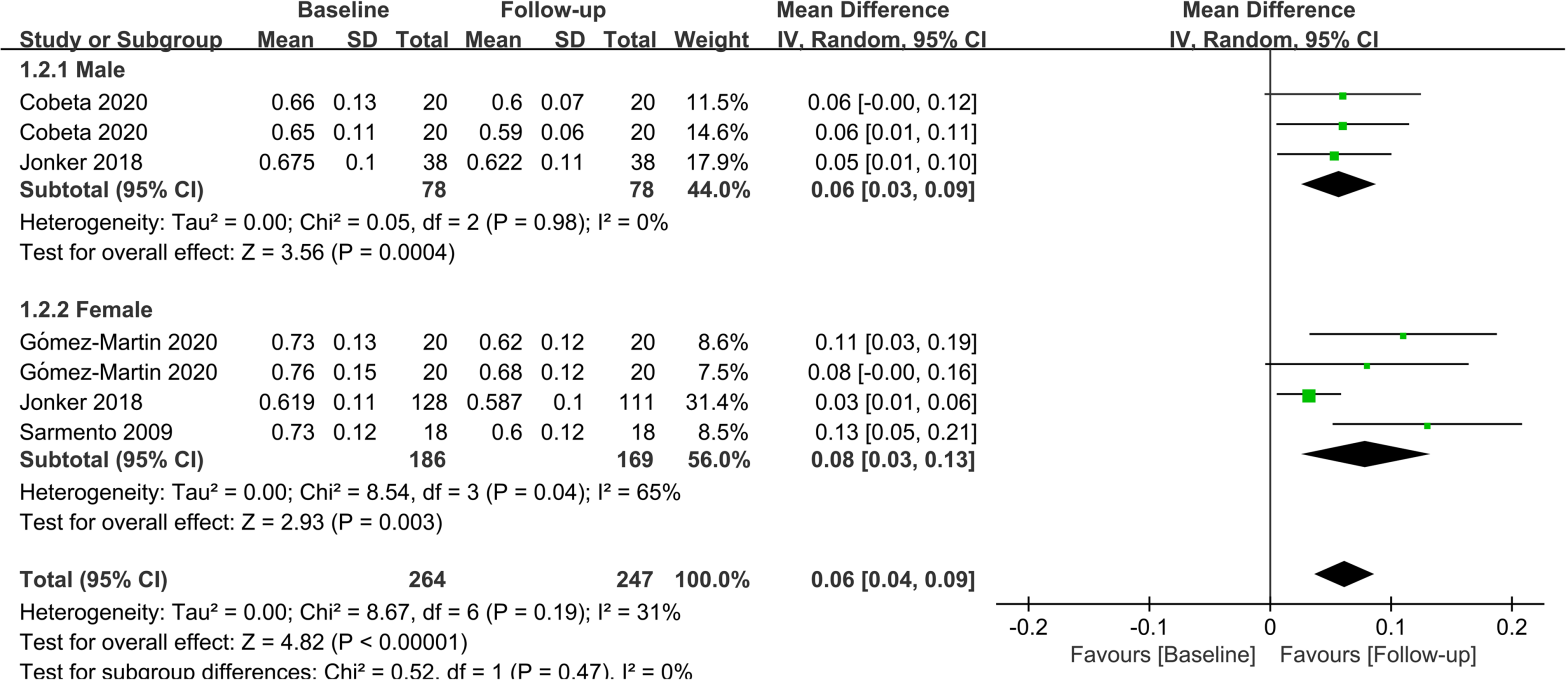
Forest plot of meta-analysis for CIMT by gender. (Cobeta 2020 and Gomez-Martin 2020 respectively included two subsets of data for meta-analysis).

In terms of gender, 2 studies investigated the effect of bariatric surgery on CIMT in men, while 3 studies evaluated the effect of bariatric surgery on CIMT in women. The combined results showed that bariatric surgery significantly reduced CIMT in both genders (Male: MD = 0.06; *P *= 0.04; Female: MD = 0.08; *P *= 0.03). The findings of the subgroup analysis regarding gender are depicted in Figure 6.

### Publication bias and sensitivity analysis

Publication bias and sensitivity analyses were performed on 6- and 12-month studies of CIMT. The results of Eggers regression test showed no publication bias (6 months: *P *= 0.172; 12 months: *P *= 0.462). The specific results are delineated in Supplementary Figures S1 and S2. Individual studies were excluded one by one to assess the stability of the results. The results showed that individual studies had a marginal impact on the overall effect, suggesting that the overall statistical results were stable.

## Discussion

Obesity is strongly associated with adverse cardiovascular outcomes, and studies have established that CIMT is significantly thicker in people with obesity, with each 0.1 mm increase in CIMT increasing the risk of myocardial infarction by 10%–15% and the risk of stroke by 10%–13% (36). Bariatric surgery is regarded as a safe and effective treatment for morbid obesity that can achieve weight loss by limiting gastric volume, regulating gastrointestinal hormones, and adjusting gut microbiota composition and function (37–39). Its positive effects on lipids, C-reactive protein, and other parameters have been corroborated. However, its effect on CIMT needs further exploration. Based on the included 23 studies, the results of this meta-analysis showed that CIMT continued to improve following bariatric surgery compared to baseline, signifying that bariatric surgery is an effective means to reduce CIMT in people with obesity. In addition, the results signaled that the benefits of bariatric surgery on CIMT increased with time. Subgroup analysis of the type of surgery demonstrated that both LRYGB and LSG were effective in reducing CIMT, while the former appeared to be more effective. By the way, the effect of bariatric surgery on CIMT was independent of gender.

The mechanism by which bariatric surgery reduces CIMT may be multifaceted. Firstly, patients lose weight and enhance their metabolism following bariatric surgery, thereby effectively improving blood lipids and subsequently decreasing CIMT (40). Secondly, the relief of complications such as hypertension can improve intimal injury and reduce blood lipid accumulation (41). Thirdly, there is a large body of evidence suggesting that bariatric surgery improves the inflammatory status of people with obesity, and can effectively regulate the levels of C-reactive protein, interleukin-6, adipokine, and other mediators, which are closely related to improvements in postoperative CIMT (42). Fourthly, fluctuations in the brain-gut endocrine axis after weight loss surgery can significantly modulate body metabolism and improve blood lipids and other parameters (43, 44). Finally, studies have shown that bariatric surgery can play a pivotal role in mediating coagulation and reducing the hypercoagulable state of blood vessels (45).

Significant improvements were noted, given that CIMT decreased by 0.09 mm, 0.12 mm, and 0.14 mm at 6 months, 12 months, and more than 18 months after the operation, respectively. From the numerical changes, it can be found that CIMT can continue to benefit after bariatric surgery. We speculate that this is inextricably linked to achieving long-lasting and effective weight loss after bariatric surgery. Presently, bariatric surgery has been a very mature means of weight loss, coupled with psychological or behavioral intervention, patients with postoperative anxiety, depression, emotional eating and overeating less and less, patients with better overall compliance, less weight rebound, so that CIMT can continue to benefit. However, a study following up on patients for five years found that CIMT was comparable to baseline levels after five years (32). Notably, the baseline CIMT in this study was only 0.57 mm, which may be a limitation for further reduction of CIMT. Furthermore, the study included a small sample size and five-year follow-up data from 62 individuals. In a word, studies on the long-term effects of bariatric surgery on CIMT are limited, with a limited sample size and high heterogeneity. Long-term outcomes of CIMT following bariatric surgery should be further investigated in larger sample-size studies.

LSG and LRYGB are the most widely used procedures for weight loss. The former achieves weight loss by excising the greater curvature to limit gastric capacity, whereas the latter achieves weight loss by altering the route of food through the digestive tract. A subgroup analysis was performed to investigate the benefits of different surgical procedures, and the results exposed that LRYGB appeared to be associated with superior benefits in CIMT in people with obesity compared to LSG, which is contrary to previous findings. However, it should be noted that in Tannaz ‘s study, CIMT decreased 0.114 mm and 0.109 mm after LSG and LRYGB, respectively, with a difference of only 0.05 mm (46). Therefore, we believe it seems less rigorous to conclude directly that LSG was superior to LRYGB. A meta-analysis conducted by Hu et al. showed that LRYGB was more effective than LSG in comorbidity resolution in the short term (47). Likewise, a meta-analysis by Gu et al. involving 9,038 participants found that LRYGB was more effective in improving long-term complications and weight loss (48). Collectively, LRYGB had better outcomes in terms of weight reduction and improving comorbidities, both in the long-term and short-term, which we hypothesize may be a plausible explanation for the above results. Therefore, LRYGB may be a better option for weight loss patients with high cardiovascular risk factors.

Previous studies have shown significant gender disparities in weight loss and a higher incidence of carotid plaque formation in men than women. Therefore, the effect of bariatric surgery on CIMT must take gender into account. Physiologically, testosterone levels are higher in men, whereas estrogen levels are higher in women. The former is a key hormone and plays an essential role in fatty acid metabolism and glycemic control (49), whilst the latter exerts significant cardioprotective and anti-inflammatory effects and can prevent endothelial apoptosis and necrosis (50, 51). Besides, there are also differences in basal metabolism between the two genders. Psychologically, women have higher requirements for their stature management and a higher compliance rate after surgery. They can better adhere to the postoperative plan and control their dietary intake, which is crucial for preventing weight rebound. Based on the aforementioned factors, a subgroup analysis was performed regarding gender, and the results revealed that bariatric surgery had similar effects on CIMT improvement in both men and women.

The strengths of our study were a large population base and detailed subgroup analysis. In contrast to previous meta-analyses, we analyzed the effect of gender on the effect of bariatric surgery for the first time. Meanwhile, a more exact time node was formulated in terms of follow-up time, and the results showed that CIMT had significantly decreased at 6 months and continued to benefit at 12 months and more than 18 months, indicating that the benefit of bariatric surgery for CIMT was durable. The limitations of the study should be considered. To begin, although the results of this study were statistically significant, they were highly heterogeneous and should be interpreted with caution. The sources of heterogeneity are manifold. Patients' inclusion criteria varied across studies, as did the degree of vascular abnormalities at baseline. At the same time, the different types of surgery are also sources of potential bias. However, subgroup analyses were performed to assess effect size across the type of surgery, and the study found that both LSG and LRYGB were effective in reducing CIMT. In addition, the site for measuring CIMT is also an important factor contributing to heterogeneity, and CIMT measured at different sites varies. It has been reported that the predilection sites for plaque formation are at the carotid bifurcation and the internal carotid artery, and measuring CIMT at these sites seems to provide a more accurate estimation of the benefits of bariatric surgery. Secondly, the small sample size of some studies lowered the accuracy of the results. Thirdly, the limited sample size restricted further subgroup analysis (e.g., age and BMI). Fourthly, it is unknown about the participants' postoperative treatment, which may affect the reliability of the results to some extent. Finally, the included studies were all observational studies and and inherently generated bias. Therefore, more comprehensive, high-quality studies are needed to validate our results.

## Data Availability

The original contributions presented in the study are included in the article/**Supplementary Material**, further inquiries can be directed to the corresponding author.

## Conclusion

In conclusion, bariatric surgery reduced CIMT in people with obesity and had a sustained, gender-independent effect. Moreover, LRYGB may be more effective than LSG in lowering CIMT.
