# Supplementary material for: Effect of bariatric surgery on carotid intima-media thickness: A meta-analysis based on observational studies
Source: Front Surg. 2023 Jan 10;9:1068681. doi: 10.3389/fsurg.2022.1068681 (PMC9871789; doi:10.3389/fsurg.2022.1068681)
Supplement: Supplementary file 1 [file Table1.docx]

**Supplementary Table 1.** Quality assessment of studies included.

| Author, year,  Study (Observational) | **Selection (Out of 4)** | | | | **Comparability**  **(Out of 2)** | **Outcomes(Out of 3)** | | | **Total**  **(Out of 9)** |
| --- | --- | --- | --- | --- | --- | --- | --- | --- | --- |
|  | Representativeness of exposed cohort | Selection of nonexposed cohort | Ascertainment  of exposure | Outcome not present at the start of the study |  | Assessment of outcomes | Length of follow-up | Adequacy of follow-up |  |
| Altin, 2018 | 1 | 1 | 1 | 1 | 1 | 1 | 0 | 1 | 7 |
| Baykara, 2018 | 1 | 1 | 1 | 1 | 1 | 1 | 1 | 1 | 8 |
| Borzì , 2020 | 1 | 1 | 1 | 1 | 1 | 1 | 1 | 1 | 8 |
| Cekici, 2021 | 1 | 1 | 1 | 1 | 1 | 1 | 0 | 1 | 7 |
| Chen, 2017 | 1 | 1 | 1 | 1 | 1 | 1 | 1 | 1 | 7 |
| Cobeta, 2020 | 1 | 1 | 1 | 1 | 1 | 1 | 0 | 1 | 7 |
| Elitok, 2020 | 1 | 1 | 1 | 1 | 1 | 1 | 1 | 1 | 8 |
| Elkan, 2020 | 1 | 1 | 1 | 1 | 1 | 1 | 0 | 1 | 7 |
| Garcia, 2013 | 1 | 1 | 1 | 1 | 1 | 1 | 1 | 1 | 8 |
| Gómez-Martin, 2020 | 1 | 1 | 1 | 1 | 1 | 1 | 1 | 1 | 8 |
| Habib, 2009 | 1 | 1 | 1 | 1 | 1 | 1 | 1 | 0 | 7 |
| Jonker, 2018 | 1 | 1 | 1 | 1 | 1 | 1 | 1 | 1 | 8 |
| Kaul, 2021 | 1 | 1 | 1 | 1 | 1 | 1 | 1 | 1 | 8 |
| Kaya, 2021 | 1 | 1 | 1 | 1 | 1 | 1 | 1 | 1 | 8 |
| Nabavi, 2022 | 1 | 1 | 1 | 1 | 1 | 1 | 0 | 1 | 7 |
| Saleh, 2012 | 1 | 1 | 1 | 1 | 2 | 1 | 0 | 1 | 8 |
| Salman, 2021 | 1 | 1 | 1 | 1 | 2 | 1 | 1 | 1 | 9 |
| Sarmento, 2009 | 1 | 1 | 1 | 1 | 1 | 1 | 1 | 0 | 7 |
| Solmaz, 2016 | 1 | 1 | 1 | 1 | 1 | 1 | 1 | 0 | 7 |
| Sturm, 2009 | 1 | 1 | 1 | 1 | 2 | 1 | 1 | 1 | 9 |
| Tschoner, 2013 | 1 | 1 | 1 | 1 | 1 | 1 | 1 | 0 | 7 |
| Yavuz, 2021 | 1 | 1 | 1 | 1 | 2 | 1 | 1 | 0 | 8 |
| Yorulmaz, 2016 | 1 | 1 | 1 | 1 | 1 | 1 | 0 | 1 | 7 |

The observational studies were assessed by the Newcastle-Ottawa Quality Assessment Scale.
