# Supplementary figures and images for: Effect of bariatric surgery on carotid intima-media thickness: A meta-analysis based on observational studies
Source: Front Surg. 2023 Jan 10;9:1068681. doi: 10.3389/fsurg.2022.1068681 (PMC9871789; doi:10.3389/fsurg.2022.1068681)

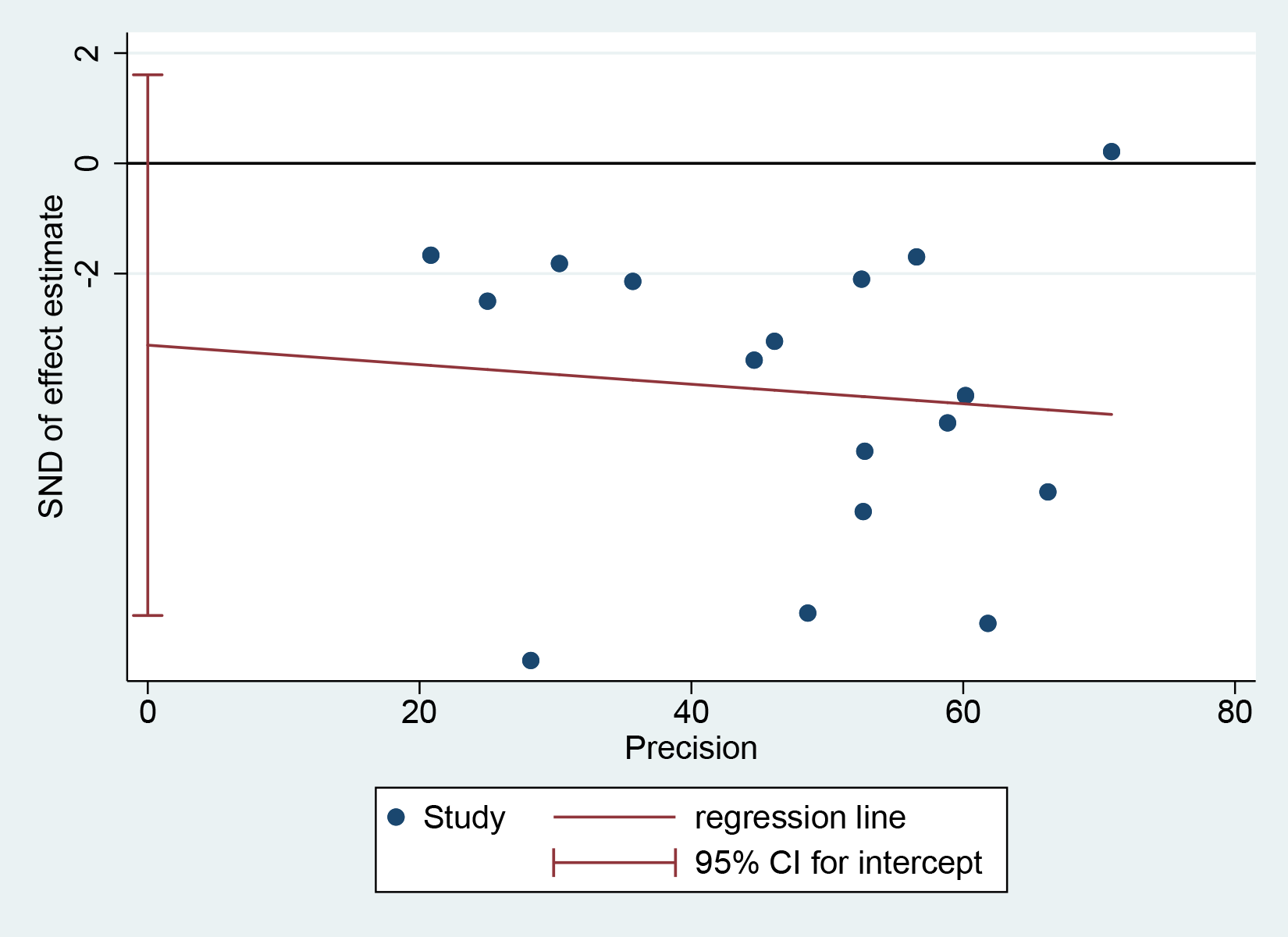

Supplement: Supplementary file 2 [file Image1.tif]

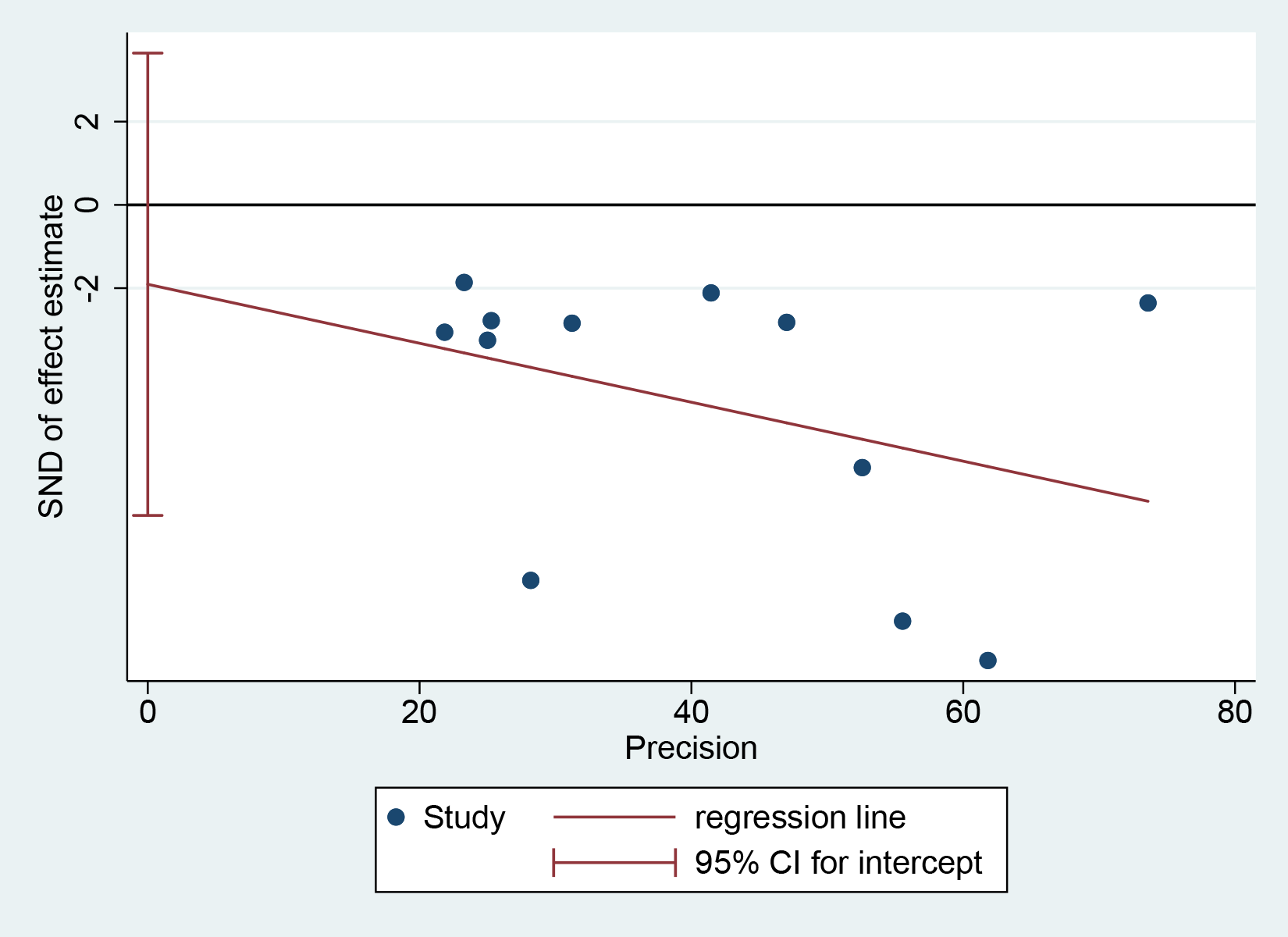

Supplement: Supplementary file 3 [file Image2.tif]
